# Are Sample Sizes Clear and Justified in RCTs Published in Dental Journals?

**DOI:** 10.1371/journal.pone.0085949

**Published:** 2014-01-21

**Authors:** Despina Koletsi, Padhraig S. Fleming, Jadbinder Seehra, Pantelis G. Bagos, Nikolaos Pandis

**Affiliations:** 1 Department of Orthodontics, School of Dentistry, University of Athens and Private Practice in Athens, Greece; 2 Barts and The London School of Medicine and Dentistry, Institute of Dentistry, Queen Mary University of London, London, United Kingdom; 3 Department of Orthodontics, Kings College Hospital NHS Foundation Trust, London, United Kingdom; 4 Department of Computer Science and Biomedical Informatics, University of Thessaly, Lamia, Greece; 5 Department of Orthodontics and Dentofacial Orthopedics, Dental School/Medical Faculty, University of Bern, Switzerland and Private Practice in Corfu, Greece; University Medical Center Göttingen, Germany

## Abstract

Sample size calculations are advocated by the CONSORT group to justify sample sizes in randomized controlled trials (RCTs). The aim of this study was primarily to evaluate the reporting of sample size calculations, to establish the accuracy of these calculations in dental RCTs and to explore potential predictors associated with adequate reporting. Electronic searching was undertaken in eight leading specific and general dental journals. Replication of sample size calculations was undertaken where possible. Assumed variances or odds for control and intervention groups were also compared against those observed. The relationship between parameters including journal type, number of authors, trial design, involvement of methodologist, single-/multi-center study and region and year of publication, and the accuracy of sample size reporting was assessed using univariable and multivariable logistic regression. Of 413 RCTs identified, sufficient information to allow replication of sample size calculations was provided in only 121 studies (29.3%). Recalculations demonstrated an overall median overestimation of sample size of 15.2% after provisions for losses to follow-up. There was evidence that journal, methodologist involvement (OR = 1.97, CI: 1.10, 3.53), multi-center settings (OR = 1.86, CI: 1.01, 3.43) and time since publication (OR = 1.24, CI: 1.12, 1.38) were significant predictors of adequate description of sample size assumptions. Among journals JCP had the highest odds of adequately reporting sufficient data to permit sample size recalculation, followed by AJODO and JDR, with 61% (OR = 0.39, CI: 0.19, 0.80) and 66% (OR = 0.34, CI: 0.15, 0.75) lower odds, respectively. Both assumed variances and odds were found to underestimate the observed values. Presentation of sample size calculations in the dental literature is suboptimal; incorrect assumptions may have a bearing on the power of RCTs.

## Introduction

Randomized controlled trials (RCTs) are considered the gold standard for assessing the efficacy and safety of an intervention and are the bedrock of evidence-based practice in medicine and dentistry. Appropriate planning of RCTs ensures validity and precise estimation oftreatment effects [1].

To increase precision in identifying a difference between treatment modalities if such a difference exists beyond chance, an *a priori* estimation of the appropriate number of participants to be included in the trial is required [2], [3]. Additionally, given the implications of RCTs in terms of time and resources, recruitment of the appropriate number of patients is imperative [4]. Unjustifiably large numbers of participants in an RCT may risk wasting sources, or may even be unethical by exposing participants to potentially ineffective or harmful treatment. Conversely, small trials may possess insufficient power to detect a clinically significant difference, if such a difference exists [5], [6], [7], [8]. In view of these issues, provision of specific details of sample size calculations in reports of clinical trials or protocol registries is recommended in the CONSORT guidelines. This allows replication of the calculation, verification of appropriate numbers in trials, and prevention of *post hoc* decisions to reduce the initially calculated necessary sample [9], [10].

Sample size calculations are based on assumptions concerning the expected and clinically important treatment effect of the new intervention compared to the control and its variance (continuous outcomes only). Additionally, levels of type I error or ‘alpha’, and type II error (‘beta’) or power must be selected. These assumptions are either based on previously published research in the same field or are derived from a pilot study prior to the commencement of the main trial [11]. Incorrect assumptions concerning the expected treatment outcomes risks leading to either underpowered studies or studies that are unnecessarily large [12]. Type I error (‘alpha’) is usually set at .05 (or less frequently at .01) and refers to the probability of 5% (or 1%) of observing a statistically significant difference between the treatment arms when no such difference exists (false positive). Type II error on the other hand is typically set at .2 (or .1) and refers to the probability of not identifying a difference if one exists (false negative). Type II error is more often expressed in terms of power (1-beta) set at 80% or 90%. Power indicates the probability of observing a difference between treatment arms if such a difference exists. Investigators are more tolerant of false negatives than false positives; this is reflected in the difference in what is considered an acceptable level for Type I (5%) and Type II errors (10% or 20%). Allowance for false positive and false negative results is unavoidable permitting reasonable sample sizes, as having statistical power of 100% would necessitate an infinite number of participants [5], [13].

Despite the importance of sample size calculations during trial design, relatively little attention has been given to the assessment of their veracity in either the medical and dental literature. A relatively recent review based on six high impact factor medical journals revealed that sample size calculations are inadequately reported and often based on inaccurate assumptions [3]. Suboptimal reporting has also been found in studies published within dentistry [14], [15], [16], [17], [18], [19], [20], [21]. In addition to lack of reporting or incomplete reporting of sample size calculations, further issues include whether the recruited numbers are calculated correctly based on the preset assumptions, and whether those *a priori* assumptions hold for the observed results [3]. It is known that sample size calculation assumptions can be doctored to approximate the available sample size rather than being truly based on the correct assumptions. In particular, setting unrealistically large treatment effects, low variances and low power will result in artificially low sample size requirements. This pattern has been highlighted for continuous outcomes published in high impact medical journals, with these studies often underpowered and predicated on optimistic assumptions [12].

The aim of the present study was to assess the quality and adequacy of sample size calculations and assumptions in RCTs published in eight leading journals in the field of dentistry over the past 20 years, to verify the accuracy of these calculations and to compare the initial assumptions with the observed values. A secondary aim was to investigate on an exploratory basis factors associated with correct performance of sample size calculations in dental specialty journals.

## Materials and Methods

The archives of eight leading dental specialty and general audience journals with the highest impact factor were screened for reports of RCTs over the last 20 years (1992–2012), by three authors (DK, JS, PSF):

*- American Journal of Orthodontics and Dentofacial Orthopedics (AJODO)*

*- British Journal of Oral and Maxillofacial Surgery (BJOMS)*

*- International Journal of Prosthodontics (IJP)*

*- Journal of Clinical Periodontology (JCP)*

*- Journal of Endodontics (JE)*

*- Pediatric Dentistry (PD)*

*- Journal of the American Dental Association (JADA)*

*- Journal of Dental Research (JDR)*

Journals were searched electronically using the terms “randomized” or “randomised” in all fields and titles and abstracts were screened for potential inclusion by two authors (DK, PSF). All types of trial design were considered including parallel with two or more arms, split-mouth, crossover, cluster, factorial and non-inferiority.

Full-text versions of the selected papers with any relevant additional supplementary material providing details of trial methodology and sample size calculation were assessed. Data abstraction forms were developed and two authors (DK, PSF) were calibrated by piloting 20 selected articles. For each paper all details contributing to sample estimation were recorded including: Type I error (alpha), power, assumptions in the interventions and control groups relating to the outcome under investigation (mean and standard deviation of difference for continuous outcomes, proportions or rate of events and difference for dichotomous and time to event outcomes). The target sample size as indicated by the *a priori* sample size calculation, the number of participants recruited and lost to follow up, and the number of studies presenting clustering effects accounted for during sample size calculation was also recorded. Finally, where applicable, the assumed variances used for sample size calculation and observed variances after completion of the study were recorded for continuous outcomes. Assumed and observed Odds Ratios (ORs) and Hazard ratios (HRs) were recorded for dichotomous and time-to-event outcomes, respectively. The following additional characteristics were also recorded for each study: number of authors, geographical region of the first author (Europe, Americas or Asia/Other), publication year, single or multi-center study, methodologist involvement, and statistical significance of the result. Collaboration with a methodologist was determined by the affiliation information given for the authors and also if explicitly stated in the “Methods” section of the study.

For each study displaying sufficient details to allow replication of the sample size calculation, calculations were repeated. To be included in the subgroup of studies that allowed for recalculation, complete reporting of type I error, one or two tailed test, power, and assumptions for the intervention and the control groups were deemed necessary (ie mean and standard deviation of difference for continuous outcomes and proportions/rate of events and difference for dichotomous/time to event outcomes). Where only type I error was not provided, an alpha level of .05 on a two-tailed test were inferred. The calculations were replicated with statistical software using the *sampsi*, *stpower, fpower* and *sampclus* family of commands where necessary (Stata 12.1, Statacorp, College Station, TX, USA). The standardized difference (%) between the actual and the estimated sample size was calculated following the formula: 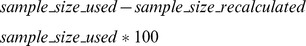



The standardized difference (%) between assumed and observed square root of variances for continuous outcome estimates was calculated accordingly: 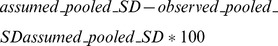



The ratio of odd ratios (ROR) of the assumed vs. the observed ORs or HRs for binary and time-to-event outcome estimates was also calculated, allowing the degree of under- or over-estimation of the required sample size to be quantified.

### Statistical Analysis

Descriptive statistics were performed for the total number of RCTs identified in each journal, geographical area, and the remaining study characteristics. Initially, Pearson chi-squared test was used to determine the association between sufficient reporting of sample size calculation, and trial characteristics including journal of publication, continent of publication, number of authors, trial design, methodologist involvement, number of research centers and arms, and significance of the results. Univariable and multivariable logistic regression modeling was used to determine the association between the feasibility of sample size calculation and predictor variables including journal, methodologist involvement, number of research centers and year of publication).The model fit was assessed using the Hosmer-Lemeshow test. In the logistic regression analysis, the journal PD was excluded as no studies with sufficient information to allow sample size recalculation were reported in this journal. All statistical analyses were conducted with statistical software (Stata 12.1, Stata Corp, College Station, TX, USA).

## Results

**Figure 1:**
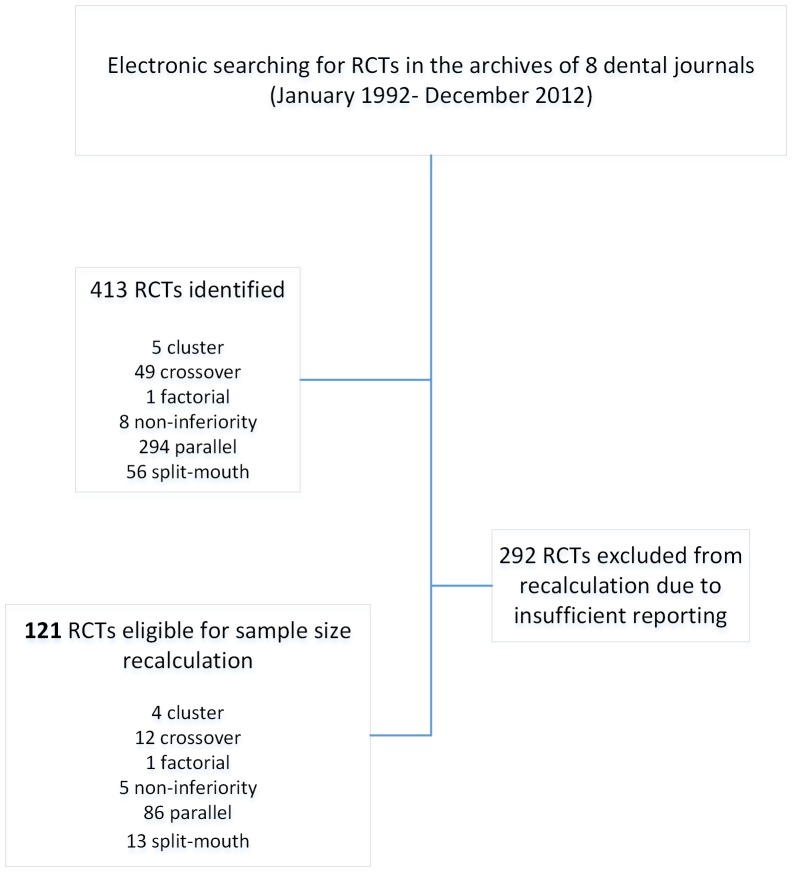
Flowchart of included studies.

Four hundred and thirteen RCTs were identified in eight leading dental specialty journals (Table 1, Figure 1). The highest number of RCTs identified was published in JCP followed by JE, AJODO and JDR. The majority of studies were conducted in a single center, by European researchers without the involvement of a methodologist. Two-arm parallel trials were most frequent, and a slight majority of studies reported statistically significant primary outcomes, whilst most studies involved 5 to 7 authors.

**Table 1:** Characteristics of the identified RCTs (n = 413).

	Sample size adequately reported		Sample size inadequately reported		Total		p-value
Journal	No.	%	No.	%	No.	%	
*AJODO*	45	15	16	13	61	15	<0.001*
*BJOMS*	37	13	1	1	38	9	
*IJP*	26	9	6	5	32	8	
*JCP*	63	22	64	53	127	31	
*JE*	55	19	14	12	69	17	
*PD*	16	5	0	0	16	4	
*JADA*	25	9	5	4	30	7	
*JDR*	25	9	15	12	40	10	
**Continent**							
*Europe*	134	46	67	55	201	49	0.16*
*Americas*	111	38	35	29	146	35	
*Asia/other*	47	16	19	16	66	16	
**No. authors**							
*1–4*	127	43	29	24	156	38	<0.001*
*5–7*	131	45	66	55	197	48	
*>7*	34	12	26	21	60	15	
**Trial design**							
*Cluster*	1	0	4	3	5	1	0.02*
*Crossover*	37	13	12	10	49	12	
*Factorial*	0	0	1	1	1	0	
*Non-inferiority*	3	1	5	4	8	2	
*Parallel*	208	71	86	71	294	71	
*Splitmouth*	43	15	13	11	56	14	
**Methodologist involvement**							
*No*	245	84	83	69	328	79	<0.001*
*Yes*	47	16	38	31	85	21	
**Center**							
*Single Center*	251	86	90	74	341	83	<0.01*
*Multi Center*	41	14	31	26	72	17	
**Significance**							
*No*	140	48	53	44	193	47	0.44*
*Yes*	152	52	68	56	220	53	
**No. arms**							
*2*	238	82	103	85	341	83	0.21*
*3*	42	14	10	8	52	13	
*4*	8	3	6	5	14	3	
*5*	2	1	1	1	3	1	
*6*	2	1	0	0	2	0	
*8*	0	0	1	1	1	0	
**Total**	**292**	**100**	**121**	**100**	**413**	**100**	

*Pearson chi^2^.

Sufficient data to allow replication of the sample size calculation was provided in 121 (29.3%) RCTs (Table 2). Most of the studies pre-specified a power of 80% to correctly identify a difference if one existed (n = 80, 66%), while in 24 studies (20%) power was set at 90%. The cut-off point for a false positive result was 5% (alpha = .05) in almost all of the studies included (n = 116/121). With regard to the type of the outcome under evaluation, continuous outcomes predominated (n = 92/121, 76%), followed by binary outcomes (n = 23/121, 19%), with few studies considering time-to-event or ordinal outcomes.

**Table 2:** Alpha level, power level and type of outcome for RCTs where recalculation of the sample sizes were feasible (n = 121).

	Recalculation Feasible	
Alpha (%)	No.	%
*0.01*	3	2
*0.025*	2	2
*0.05*	109	90
*Inferred 0.05*	7	6
**Power (%)**		
*75*	1	1
*80*	80	66
*82*	1	1
*85*	5	4
*86*	2	2
*90*	24	20
*94*	1	1
*95*	6	5
*99*	1	1
**Outcome**		
*Binary*	23	19
*Continuous*	92	76
*Time to event*	4	3
*Ordinal*	2	2
**Total**	**121**	**100**

The standardized percentage difference between sample size used and recalculated was determined in the 121 RCTs that allowed for replication of the calculations (Table 3). The overall median discrepancy ranged from −237.5% to 84.2%, with a median value of 15.2% (IQR = 35); positive values indicate that the sample size recruited exceeded requirements based on *a priori* assumptions. The subgroups of studies that overestimated, underestimated or correctly calculated the sample size required, based on *a priori* assumptions are also presented in Table 4.

**Table 3:** Median, range and interquartile range for standardized percentage difference where sample recalculation was feasible (n = 121).

	N	median	min	max	IQR
**Journal**					
*AJODO*	16	3.5	−93.3	45.0	38.5
*BJOMS*	1	19.3	19.3	19.3	0.0
*IJP*	6	22.2	−53.5	67.6	85.6
*JCP*	64	19.1	−237.5	61.9	30.7
*JE*	14	11.3	−73.3	84.2	23.7
*JADA*	5	46.7	−66.0	65.5	16.6
*JDR*	15	15.2	−232.2	72.2	40.0
**Continent**					
*Europe*	67	12.5	−237.5	84.2	33.3
*Americas*	35	12.9	−73.3	72.2	51.5
*Asia/other*	19	20.0	−140.0	67.6	29.1
**No. authors**					
*1–4*	29	4.0	−237.5	84.2	67.4
*5–7*	66	19.3	−132.3	67.6	27.3
*>7*	26	13.8	−232.2	72.2	28.8
**Trial design**					
*Cluster*	4	4.3	−11.1	22.6	30.7
*Crossover*	12	28.2	3.2	84.2	41.3
*Factorial*	1	26.4	26.4	26.4	0.0
*Non-inferiority*	5	−4.9	−232.2	25.3	37.6
*Parallel*	86	9.1	−237.5	72.2	38.8
*Splitmouth*	13	48.4	−80.0	65.5	31.3
**Methodologist involvement**					
*No*	83	19.4	−237.5	84.2	40.0
*Yes*	38	4.5	−232.2	46.1	39.6
**Center**					
*Single Center*	90	12.7	−237.5	84.2	40.1
*Multi Center*	31	18.2	−232.2	67.6	21.9
**Significance**					
*No*	53	19.4	−237.5	72.2	32.1
*Yes*	68	12.5	−232.2	84.2	40.3
**No. arms**					
**2**	103	14.3	−237.5	84.2	35.0
**3**	10	10.2	−132.3	58.3	44.6
**4**	6	30.7	−42.9	55.6	88.4
**5**	1	4.2	4.2	4.2	0.0
**8**	1	26.4	26.4	26.4	0.0
**Total**	**121**	**15.2**	**−237.5**	**84.2**	**35.0**

**Table 4:** Standardized percentage difference per subgroup for RCTs where recalculation of the sample sizes was feasible (n = 121).

	Standardized difference		
Subgroup	Recalculated>actual* n = 35	Recalculated<actual** n = 84	Recalculated = actual n = 2
median	−30.00	23.60	0.00
min	−237.50	1.60	0.00
max	−1.70	84.20	0.00
IQR	53.35	22.58	0.00
			

*Recalculated > actual indicates underestimation of the sample size by the authors of the RCTs and is characterized by negative values for the calculated standardized difference.

**Recalculated < actual indicates overestimation of the sample size by the authors of the RCTs and is characterized by positive values for the calculated standardized difference.

**Figure 2:**
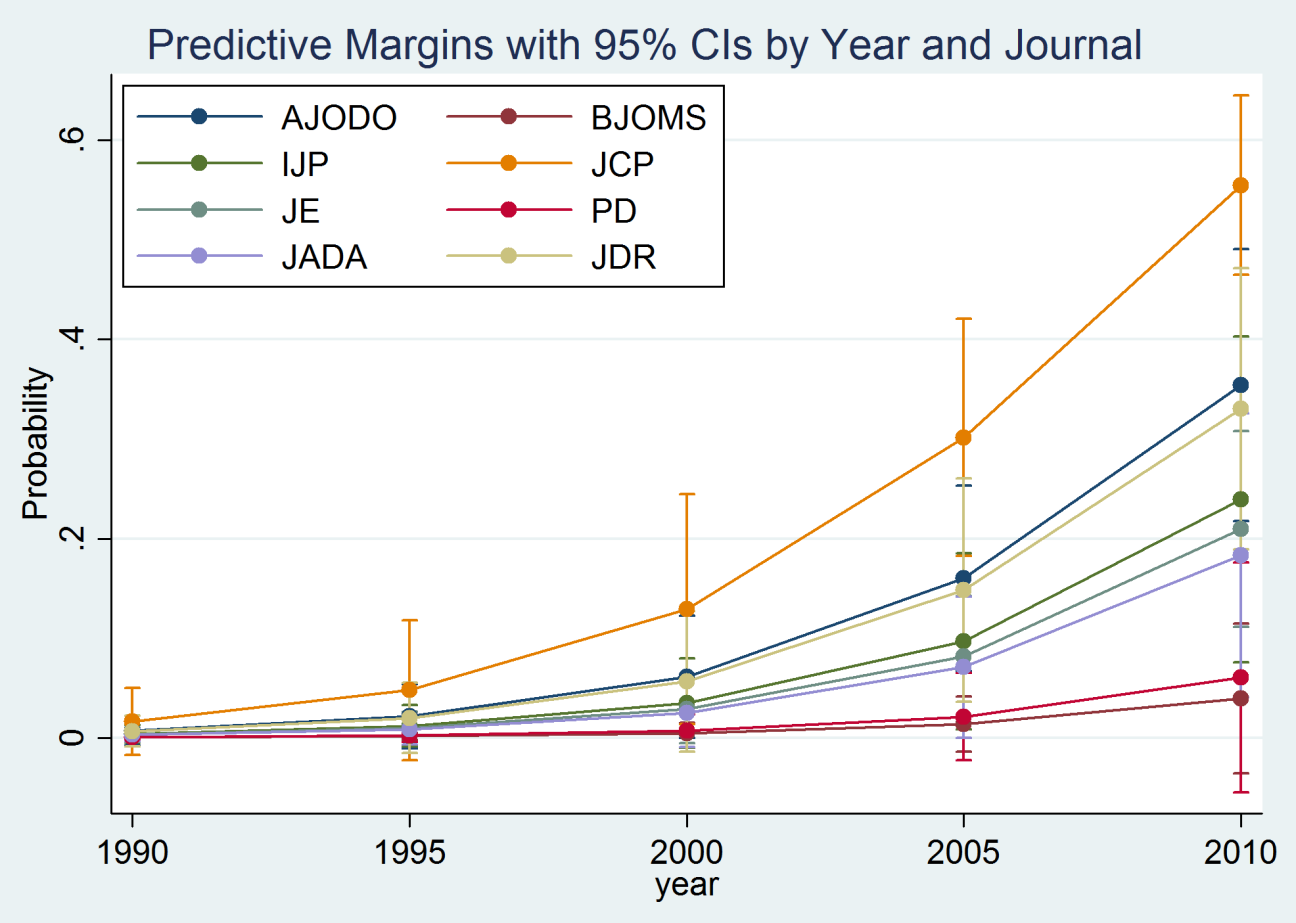
Predicted probabilities of adequate sample size reporting with 95% confidence intervals (CIs) derived from the adjusted model for reporting sample size calculation details based on the journal and year of publication.

Multivariable logistic regression (Table 5) demonstrated that JCP had the highest odds of adequately reporting sufficient data to permit sample size recalculation followed by AJODO and JDR, with 61% (OR = 0.39, CI: 0.19, 0.80) and 67% (OR = 0.34, CI:0.15, 0.75) lower odds, respectively. The involvement of a methodologist in the statistical analysis and trial methodology of the included studies resulted in 97% higher odds (OR = 1.97, CI: 1.10, 3.53) of appropriate reporting. There was evidence that a multi-center setting also resulted in 86% higher odds of sufficient reporting to allow recalculation (OR = 1.86, CI: 1.01, 3.43). For each additional year of publication until 2012, the odds of inclusion of sample size assumptions increased by 24% (OR = 1.24, CI: 1.12, 1.38). The predicted probabilities from the adjusted model for sufficient sample calculations reporting are shown by year and journal in Figure 2. The journal PD was excluded from the logistic regression analysis, as PD did not contribute any studies with sufficient information to allow sample recalculation.

**Table 5:** Univariable and multivariable logistic regression derived ORs and 95% confidence intervals (CI) for the feasibility of sample size recalculation for the identified Randomized Controlled Trials (n = 413).

Predictor variables		Univariable analysis			Multivariable analysis		
Variable	Category/Unit	OR	95% CI	p-value	OR	95% CI	p-value
**Journal**	*AJODO*	0.35	0.18, 0.68	0.002	0.39	0.19, 0.80	0.01
	*BJOMS*	0.03	0.00, 0.20	<0.001	0.04	0.00, 0.29	0.002
	*IJP*	0.23	0.09, 0.59	0.002	0.26	0.10, 0.71	0.01
	*JCP*	Baseline (reference)					
	*JE*	0.25	0.13, 0.50	<0.001	0.26	0.13, 0.54	<0.001
	*JADA*	0.20	0.07, 0.55	0.002	0.15	0.05, 0.43	<0.001
	*JDR*	0.59	0.29, 1.22	0.16	0.34	0.15, 0.75	0.01
**Methodologist involvement**	*No*	Baseline (reference)					
	*Yes*	2.29	1.39, 3.77	0.001	1.97	1.10, 3.53	0.02
							
**Number of centers**	*Single center*	Baseline (reference)					
	*Multi center*	2.03	1.20, 3.45	0.01	1.86	1.01, 3.43	0.046
**Year**	*(per year)*	1.23	1.12, 1.35	<0.001	1.24	1.12, 1.38	<0.001

**Figure 3:**
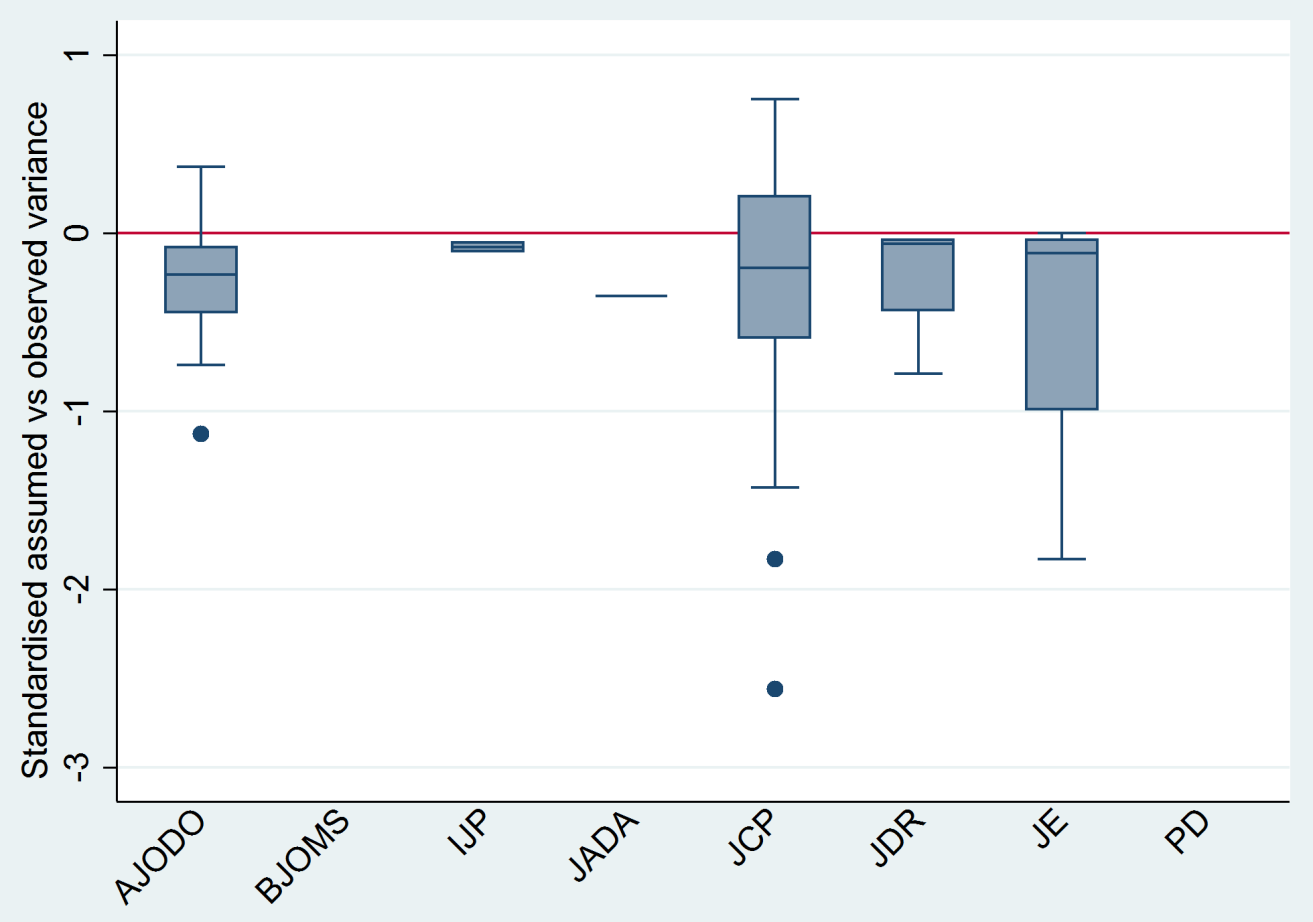
Boxplots of percentage standardized difference between assumed and observed variance for continuous outcome from the RCTs where sample size recalculation was feasible (n = 92/121) based on journal of publication. The horizontal line at zero indicates perfect agreement between assumed and observed variance. Median values below zero indicate optimistic assumptions of variance (smaller than observed) and *vice versa*.

**Figure 4:**
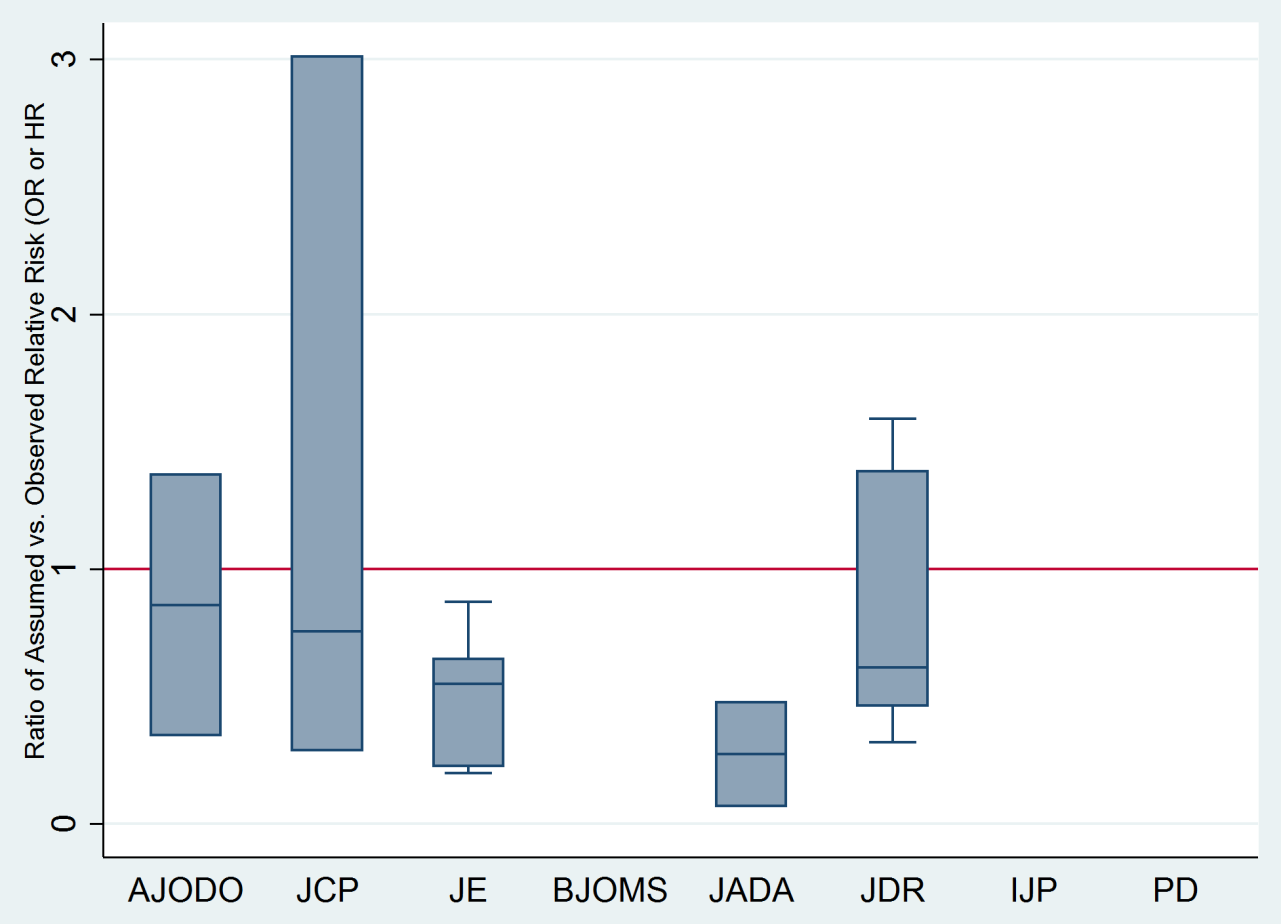
Boxplots of ratio of Odds Ratios (ORs) of assumed compared to observed ORs or HRs for binary, ordinal and time-to-event outcomes from the RCTs where sample size recalculation was feasible (n = 29/121) based on journal of publication. The horizontal line at 1 indicates perfect agreement between assumed and observed ORs. Median values below 1 indicate optimistic assumptions of variance (smaller than observed) and *vice versa*.

Finally, for continuous outcomes, the standardized difference between assumed and observed variance indicated an overall median discrepancy of 2.92% (IQR = 53.8%; Figure 3); negative median values (below zero) show overly optimistic assumption on the expected variance. As for binary and time-to-event outcomes, the overall median ratio of assumed compared to observed ORs was 0.61 (IQR = 1.01; Figure 4) with values less than 1 indicating optimistic assumptions on the expected differences in the odds of events between treatment groups.

The median number of participants required based on the sample size assumptions in the 121 included RCTs was 42 (range: 10–832), whereas the median number recruited was 50 (range:10–983) participants. A median of two dropouts per trial was recorded.

Of the 121 studies that included sufficient data concerning sample size assumptions, 71 demonstrated some sort of clustering effect for the primary outcome measure. However, only 10/121 trials accounted for the correlated nature of the data in performing the sample size calculations at the design stage; clustering was accounted for in the statistical methods in the majority of these studies (n = 53, 74.6%).

## Discussion

The present work is the first large scale study to analyze the veracity of sample size calculations of RCTs, along with other associations in leading specific and general audience dental journals. The overall median discrepancy identified between presented and recalculated sample sizes was 15.2% (−237.5%, 84.2%) after making provision for losses to follow-up, indicating a tendency to slightly over-estimate required numbers. However, this finding was based on a small portion of RCTs, as inadequate data to allow for replication of sample size assumptions was typical (70.7%). This finding mirrors a recent study in orthodontics, which highlighted that replication of calculations was possible in just 29.5% of the RCTs; the median discrepancy between presented and recalculated sample size was 5.3%, although recalculations were conducted on fewer studies (n = 41) [21]. Similarly, in biomedical research, comprehensive sample size calculations, with adequate data permitting replication was identified in 34% of studies [3] and only 19% of studies in the field of plastic surgery [22].

The multivariable logistic regression model demonstrated that articles published in JCP had the highest odds for correctly reporting assumptions for sample size calculation, followed by the AJODO and the JDR. Evidence of underpowered studies in periodontology was highlighted in the past; this may have provoked increased awareness of the necessity for clear and accurate reporting of sample size calculations within this specialty[14].

Another finding of note was the discrepancy between the assumed and observed variances and ORs for the intervention and control groups. This difference may lead to insufficiently powered trials lacking the capacity to identify treatment differences risking incorrect inferences based on inconclusive outcomes [12]. The discrepancy between the final overestimation of the sample size compared to assumptions on variance and odds of the events is related to inflation of the sample size to account for possible losses to follow-up. The main reasons for inadequate assumptions of variances are that the assumptions are based on initial piloting, with the researchers following exactly the same variances for a much larger RCT, disregarding the fact that variance is not fixed [11]. Initial piloting within each trial may help optimize sample calculations. Other methods proposed to overcome the uncertainty of nuisance parameters are “sample size reviews” or “designs with internal pilot studies” that allow recalculation of the sample size during the course of a clinical trial, with subsequent adjustments to the initially planned size [23], [24].

The recruitment of a median number of 50 participants in a dental RCT is often a realistic objective, which may reflect researchers' tendency to arrive at an achievable and feasible size of sample to test the differences between interventions for a research question. However, whether the appropriate sample size is determined from valid and pre-specified assumptions for the intervention groups can rarely be determined. It is possible that effect sizes and assumptions may be manipulated to arrive at the desired number of participants [2]. This practice has led to calls for discontinuation of the usage and reporting of sample size calculations [25].

This study also confirmed the apparent lack of reporting of specific trial characteristics, which are necessary for accurate sample size estimation. In particular, correlated data, which may contribute to clustering effects and outcomes more closely matched within clusters than between them, was poorly handled [26]. The number of studies accounting for these effects was disappointing with only a small fraction reporting sufficient information in the sample estimation assumptions.

A limitation of the present work is that outcomes were based solely on reported information from the included RCTs. Protocols of RCTs published in trial registries prior to the commencement of the study would help eliminate possible deviations from *a priori* assumptions [27]. However, protocols were rarely identified for the RCTs included in the present study.

Finally, if reporting of sample size assumptions is to continue, emphasis should be placed on encouraging researchers, authors, editors and peer reviewers to overhaul the reporting quality of submitted clinical trials prior to publication, in line with the CONSORT guidelines for clear and transparent reporting.
