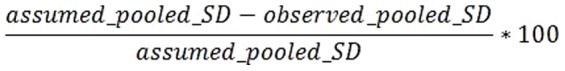# Correction: Are Sample Sizes Clear and Justified in RCTs Published in Dental Journals?

**DOI:** 10.1371/journal.pone.0093238

**Published:** 2014-03-18

**Authors:** The PLOS ONE Staff

The two equations in the Materials and Methods section are incorrect. The corrected versions are provided here.[Fig pone-0093238-g001] [Fig pone-0093238-g002]

**Figure 1 pone-0093238-g001:**
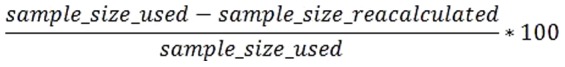


**Figure 2 pone-0093238-g002:**